# Estimating benefit equity of government health subsidy in healthcare Services in Shandong Province, China: a cross-sectional study

**DOI:** 10.1186/s12939-018-0775-3

**Published:** 2018-05-18

**Authors:** Wenzhe Qin, Lingzhong Xu, Jiajia Li, Long Sun, Gan Ding, Hui Shao, Ningze Xu

**Affiliations:** 1 Department of Social Medicine and Health Management School of Public Health, Shandong University, Road44# Jinan, Shandong, CN China; 2 Department of Global Health Systems and Development, School of Public Health and Tropical Medicine, Tulane University, New Orleans, LA USA; 3 Key Lab of Health Technology Assessment, National Health Commission of the Peoples Republic of China, School of Public Health, Fudan University, Shanghai, China

**Keywords:** Government health subsidy, Benefit incidence analysis, Equity, Healthcare service

## Abstract

**Background:**

Government health subsidy (GHS) is an effective tool to improve population health in China. Ensuring an equitable allocation of GHS, particularly among the poorer socio-economic groups, is a major goal of China’s healthcare reform.

The paper aims to explore how GHS was allocated across different socioeconomic groups, and how well the overall health system was performing in terms of the allocation of subsidy for different types of health services.

**Methods:**

Data from China’s National Health Services Survey (NHSS) in 2013 were used. Benefit incidence analysis (BIA) was applied to examine if GHS was equally distributed across income quintile. Benefit incidence was presented as each quintile’s percentage share of total benefits, and the concentration index (CI) and Kakwani index (KI) were calculated. Health benefits from three types of healthcare services (primary health care, outpatient and inpatient services) were analyzed, separated into urban and rural populations. In addition, the distribution of benefits was compared to the distribution of healthcare need (measured by self-reported illness and chronic disease) across income quintiles.

**Results:**

In urban populations, the CI value of GHS for primary care was negative. (− 0.14), implying an allocation tendency toward poor region; the CI values of outpatient and inpatient services were both positive (0.174 and 0.194), indicating allocation tendencies toward rich region. Similar allocation pattern was observed in rural population, with pro-poor tendency of primary care service (CI = − 0.082), and pro-rich tendencies of outpatient (CI = 0.153) and inpatient services (CI = 0.203). All the KI values of three health services in urban and rural populations were negative (− 0.4991,-0.1851 and − 0.1651; − 0.482, − 0.247and − 0.197), indicating that government health subsidy was progressive and contributed to the narrowing of economic gap between the poor and rich.

**Conclusions:**

The inequitable distribution of GHS in China exited in different healthcare services; however, the GHS benefit is generally progressive. Future healthcare reforms in China should not only focus on expanding the coverage, but also on improving the equity of distribution of healthcare benefits.

## Background

The enjoyment of the highest attainable standard of health without distinction of race, religion, and political belief, economic or social conditions is one of the fundamental rights of every human being [1]. In healthcare, one of the priority of national health policy is to guarantee equitable access to basic healthcare services. (Universal Health Coverage, UHC) [2]. Health equity is acknowledged as a critical component of the post-2015 sustainable development agenda and is an essential element of any country’s path towards UHC [3]. A recent World Bank Group study also found that more countries in the world had initiated UHC programs designed to expand access to health care and reduce the number of people impoverished by paying for the health care they need [4]. Beginning from 2009, a new health reform aiming to achieve UHC among all Chinese citizens was implemented in China.

Government health subsidy (GHS) is provided to pay for the healthcare expenditures incurred in public medical institutions. Patients who spent a unit of health service will receive a unit of subsidy. GHS is an effective tool to improve the national health status and achieve universal health coverage. The Chinese governmental budget for health, although below average worldwide, has been increased substantially in the last decade [5]. However, researchers have observed a consistently increasing trend of out-of-pocket (OOP) payments [6]. Access to health care became more difficult for the poor who could not afford basic healthcare [7–9]. Growing inequalities between rural and urban areas in healthcare use and health outcomes were reported [10, 11].

Chinese GHS was given to both healthcare providers and patients. Subsidies to patients mainly through a variety of medical insurances (New Rural Cooperative Medical Scheme,Urban Resident Basic Medical Insurance, and Urban Employee Basic Medical Insurance), and its benefit allocation is relatively definite. However, GHS to providers depends on the volume of healthcare services received by their patients. Socioeconomically disadvantaged population groups, despite a generally higher need, use healthcare less often than individuals with higher socioeconomic status. So the wealthy received more subsidies than the poor, resulting in a scenario that “the poor subsidize the rich” and the consequent healthcare inequity. Improving the equitable benefit allocation of GHS, particularly among the country’s lower socio-economic groups, is a major goal of China’s healthcare reform [12]. However, little is known about the impact of the recent reforms on the previously inequitable distribution of health care benefits. Thus, a proper assessment of the benefit distribution of government health subsidy among different income groups is needed in order to help policy makers develop financial risk protection strategies to ensure that the poor benefit from government health subsidies.

The paper aims to explore how GHS was allocated across different socioeconomic groups, and how well the overall health system was performing in terms of the allocation of subsidy in different types of health services.

## Methods

### Data sources

Our data were collected from China’s 5th National Health Services Survey (NHSS) conducted in Shandong provincein 2013. Stratified multi-stage random sampling was applied: in the first stage, 20 counties/districts were selected from total 137 counties as the primary sampling units (PSUs) throughout the province (which were divided into10 rural counties and 10 urban districts). From each PSU, 10 villages were selected as the secondary sampling units (SSUs). In the third stage, 60 households were randomly selected from each SSU. The descriptive and socioeconomic characteristics of each income quintile are summarized in Table 1.Finally, a total of 11,920 households, consisting of 39,032 individuals, were included in the survey. Information including socio-demographics, household expenditures, healthcare utilization of sample population, healthcare expenditures, access to health services, and other complementary data were collected.

**Table 1:** Descriptive statistics by income quintile

Region	income quintiles	Living standards(%)	Per capita expenditures	Per capita household expenditures	No.of surveyed individuals	No.of surveyed households
Urban	1 (poorest)	0.08	4592.07	11,895.16	3067	1184
	2	0.14	6243.26	20,179.86	3827	1184
	3	0.18	7723.67	26,752.33	4101	1184
	4	0.23	10,157.01	34,846.10	4062	1184
	5 (richest)	0.37	14,903.99	54,757.04	4350	1184
	subtotal				19,407	5920
Rural	1 (poorest)	0.09	3761.51	8576.25	2736	1200
	2	0.14	4464.72	13,859.25	3725	1200
	3	0.19	5397.27	18,566.60	4128	1200
	4	0.23	6371.05	23,164.09	4363	1200
	5 (richest)	0.35	8840.91	34,427.97	4673	1200
	subtotal				19,625	6000

In addition to survey data, the data for estimating per capital subsidy and healthcare utilization related data of Shandong province were obtained from two sources. Per capital GHS was calculated from the Finance Yearbook of China, China Statistical Yearbook for Regional Economy and heath care facilities’ annual financial reports.

In order to explore the differences in allocation equity, the benefit distribution of GHS was analyzed by three healthcare service types in this paper. The healthcare services were divided into primary care, outpatient and inpatient service, both in urban and rural areas. Primary care utilization included healthcare services which delivered at community health centers (CHCs), village clinics (VCs) and township health centers (THCs). Outpatient and inpatient services were delivered at county hospitals (CHs), district hospitals (DHs) and municipal hospitals (MHs).

### Measurement

#### *Socioeconomic group*s

The households were classified into quintiles, corresponding to five socioeconomic groups based on household expenditure per equivalent adult. A common approach is to define the number of adult equivalents (AE) in the household as

$$ AE={\left(A+\alpha K\right)}^{\theta, } $$

Where *A* is the number of adults in the household, *K* is the number of children (0–14 years old), α is the equivalent parameter, and θ reflects the degree of economies of scale. According to previous study, our study set α and θ to 0.5 and 0.75, respectively [13, 14].

#### Healthcare utilization

The data of healthcare utilization was collected from the survey. Outpatient visits were reported for a 2-week recall period prior to the survey and inpatient days were reported for a 12-month recall period. In order to calculate the allocation of benefits for the different quintiles of the entire population, the utilization of healthcare services in the sample population was weighted.

### Outcomes

#### Unit GHS and benefit incidence

Consider the benefit incidence of GHS for healthcare services. This is given by the equation [15]:

$$ Xj=\sum \limits_{k=1}^3{H}_{kj}\frac{S_k}{H_k} $$

Where *X*_*j*_ is the total GHS to group *j*, *H*_*kj*_ represents the number of healthcare utilization of group *j* at *k* type of healthcare service, *S*_*k*_ is government subsidies for healthcare service at type k, and *H*_*k*_is the total number of utilization of healthcare service at type *k*. Note that is the unit GHS for healthcare service at type *k* .

#### Healthcare need

We used self-reported illness and chronic disease as the indicator for healthcare need. The data included information on self-reported illness or symptoms in the previous 14 days. What’s more, people with chronic diseases were all considered to have healthcare needs.

### Data analysis

We applied BIA with concentration curve, Lorenz curve, concentration index (CI) and Kakwani index (KI) to examine the benefit equity of GHS across socio-economic groups. BIA describes the allocation of GHS across households ranked by their living standards [16–18]. Per capita household expenditure adjusted by AE is used as the measure of living standards [19].

Benefits from government spending on a service are said to be pro-poor if the concentration curve for these benefits is above the 45-degree line, and if below, benefits are said to be pro-rich [20]. If pro-poor, a negative CI will be calculated, otherwise a positive CI will be estimated from the sample. The further the curve is above the 45-degree line, the more concentrated the subsidy is amongst the poor and the higher the value of the CI, and vice versa [21].

**Fig. 1:**
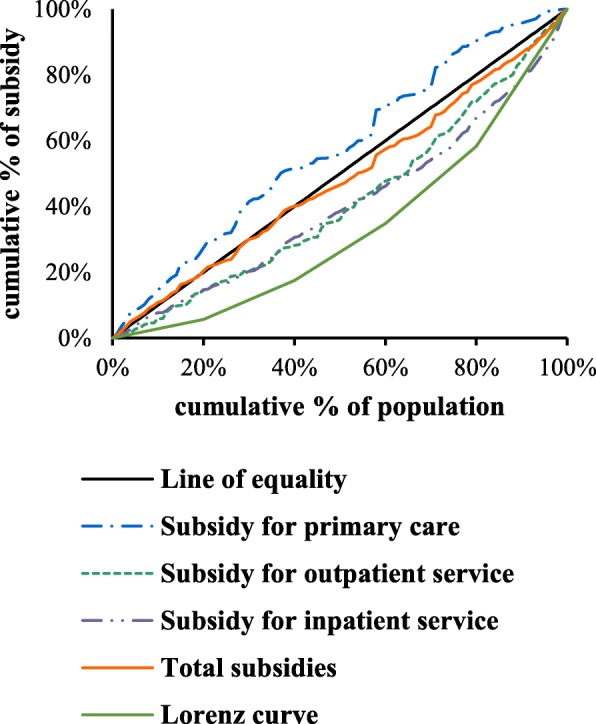
Concentration curves of GHS in the urban population. Line of equality; Subsidy for primary care; Subsidy for outpatient service; Subsidy for inpatient service; Total subsidies; Lorenz curve

The Kakwani’s progressivity index (KI) is used to measure progressivity as twice the area between the Lorenz curve and the Concentration curve for GHS (Fig. 1). The KI is calculated as:

$$ \mathrm{KI}=C\hbox{--} G, $$

where C is the concentration index and G is the Gini coefficient. The value of KI ranges from − 2 to 1.By convention, a negative number indicates progressive; a positive number indicates regressive.

Furthermore, the benefit and Lorenz curves are generated from samples rather than from the entire population, so it is important to apply statistical tests of dominance [22]. The dominance test judges whether the difference between the GHS allocation and the two benchmarks is statistically significant [23]. Multiple comparisons were performed, with null defined as indistinguishable curves [24].

## Results

Table 2 shows the allocation of GHS in the urban population used at the primary care, outpatient and inpatient services and stratified by income quintile. Concentration curves of the subsidies in the urban population at different healthcare services are shown in Fig. 1(Fig. 1 Concentration curves of GHS in the urban population.).The CI value of primary care was negative (− 0.117), suggesting that the subsidies in primary care were pro-poor with the poorest quintile getting 27.37% of all benefits as compared to 10.41% going to the richest. The two positive CI values of outpatient and inpatient services indicated that the government subsidies for outpatient and inpatient services favored the rich people significantly as shown in the CI values of 0.167 and 0.189. The CI value of total health services was positive (0.04), but appeared to be close to zero, suggesting that the GHS for all healthcare services were slightly pro-rich. All the KI values of healthcare services were negative, indicating that the GHS in all healthcare services were progressively distributed by socioeconomic status. Results showing the plots of Lorenz curve and the concentration curves are presented in Fig. 1. This provides a visual implementation of the progressivity of GHS on healthcare services.

**Table 2:** Distribution of GHS by income quintile across different service types (urban populations)

Income quintiles	Per capita household expenditure	Primary health care	Outpatient care	Inpatient care	Total
Lowest quintile	5.65%	27.37%	13.75%	14.80%	20.97%
2	11.87%	20.58%	14.09%	15.69%	18.09%
3	17.37%	19.22%	20.27%	15.96%	17.57%
4	23.41%	22.42%	24.05%	21.22%	21.81%
Highest quintile	41.70%	10.41%	27.84%	32.33%	21.56%
Gini/CI (SE)	0.359(0.01)	-0.117^a^(0.05)	0.167^a^(0.04)	0.189^a^(0.03)	0.04(0.03)
Kakwani index		−0.476	−0.192	− 0.17	− 0.319
Dominance test					
-against45°line		D^+^	D^−^	D^−^	None
-against Lorenz curve		D^+^	D^+^	None	D^+^

Note: ^a^significant at 0.05

“None” indicates failure to reject the null hypothesis that curves are indistinguishable at the 5% significance level

D+/D- indicates pro-poor/ pro-rich

**Fig. 2:**
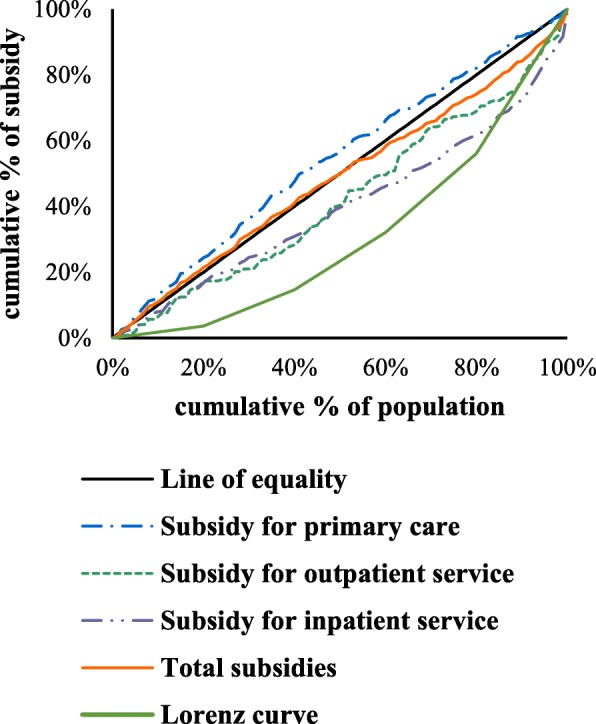
Concentration curves of GHS in the rural population. Line of equality; Subsidy for primary care; Subsidy for outpatient service; Subsidy for inpatient service; Total subsidies; Lorenz curve

In rural population, the distribution of GHS used at the primary care, outpatient and inpatient services and stratified by income quintile is shown in Table 3. Concentration curves of GHS in the urban population at different healthcare services are shown in Fig. 2 (Fig. 2 Concentration curves of GHS in the rural population). Similarly to urban people, the CI value of GHS in primary care was significantly negative (− 0.079), and no notable evidence of inequality was found. This result indicates that the poor received a greater proportion of GHS than the rich when they sought primary care. In outpatient and inpatient services, the results were just the opposite. The GHS for outpatient and inpatient services are pro-rich with statistically positive CI values (0.137 and 0.203), suggesting that a relatively higher healthcare subsidies were allocated to the wealthy. On the other hand, the GHS for all healthcare services were progressive with all negative KI values.

**Table 3:** Distribution of GHS by income quintile across different service types (rural populations)

Income quintiles	Per capita household expenditure	Primary health care	Outpatient care	Inpatient care	Total
Lowest quintile	3.67%	23.95%	17.34%	16.87%	21.18%
2	10.97%	23.76%	12.12%	13.80%	19.86%
3	17.51%	18.06%	22.17%	15.50%	17.06%
4	23.88%	16.12%	17.48%	15.07%	15.71%
Highest quintile	43.97%	18.12%	30.89%	38.76%	26.18%
Gini/CI (SE)	0.4(0.03)	− 0.079^a^(0.03)	0.137^a^(0.06)	0.203^a^(0.04)	0.032^a^(0.03)
Kakwani index		−0.479	− 0.263	− 0.197	−0.368
-against45°line		None	D^−^	D^−^	None
-against Lorenz curve		D^+^	None	None	None

Note: ^a^significant at 0.05

“None” indicates failure to reject the null hypothesis that curves are indistinguishable at the 5% significance level

D+/D- indicates pro-poor/ pro-rich

**Fig. 3:**
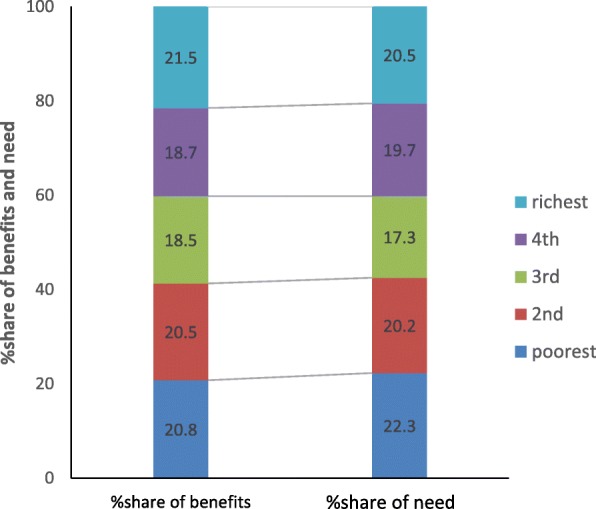
Distribution of healthcare benefits from all healthcare services in relation with healthcare need in urban populations. Poorest; 2nd; 3rd; 4th; richest

**Fig. 4:**
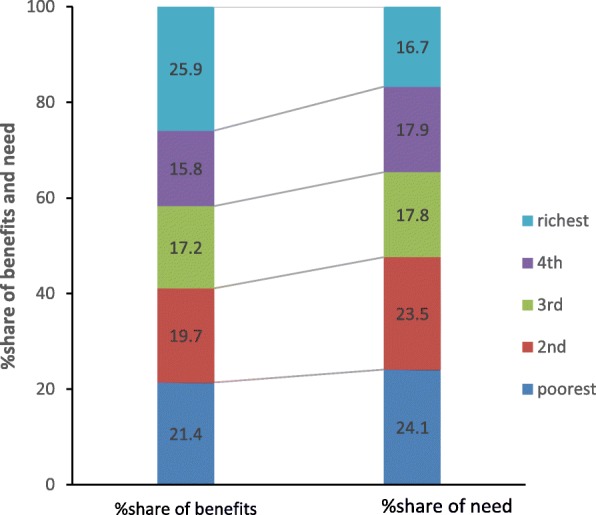
Distribution of healthcare benefits from all healthcare services in relation with healthcare need in rural populations. Legend: Poorest; 2nd; 3rd; 4th; richest

Allocations of health benefits in relation to need for healthcare across five socioeconomic groups in urban and rural are presented in Fig. 3 (Fig. 3 Distribution of healthcare benefits from all healthcare services in relation with healthcare need in urban populations) and Fig. 4 (Fig. 4 Distribution of healthcare benefits from all healthcare services in relation with healthcare need in rural populations). In urban populations, distributions of healthcare need measured by “self-reported illness and chronic disease” is essentially balanced with allocations of health benefits among five groups. In rural population, however, Fig. 4 shows that, the poorest group accounted for 24.11% of total healthcare need, but accrued only 21.4% of total healthcare benefits. On the contrary, the richest group while was in need of 16.7% healthcare utilized 25.88% of total benefits. Observations across rural groups showed that the need for healthcare reduced, but health benefits increased with better socioeconomic position, which demonstrates the inequitable allocation of GHS in rural populations.

## Discussion

In the global perspective, most of the countries are committed to take strategies or reform to achieve UHC. Health financing arrangements are central for achieving UHC, as it is through these mechanisms that resources are raised, financial risks and barriers to access are minimized, and services are purchased in ways that promote efficiency, eliminate waste and reduce inequalities in coverage. For the NHS countries, such as Britain and Malaysia, basic healthcare services are funded mainly through taxes and provided to residents free of charge. Therefore, these countries have achieved the goals of population coverage and universal financial protection. For the SHI countries, such as China, Thailand and Germany, the government increased funding as well as health fiscal subsidies to purchase services [25]. The large informal sector is a major challenge to the extension of population coverage in SHI countries that must seek the optimal mix of tax subsidy and health insurance for universal coverage [26]. Other countries, such as the United States and South Africa, rely on market mechanisms to achieve population disease risk protection so that the government has a weak role in the basic medical insurance system.

Since 2009, the Government of China initiated health-care reforms to achieve universal access to health care by 2020 [27]. As a result, the central governmental budget for health, has been increased substantially. So, to what extent has a country progressed toward Universal Health Coverage? The benefit equity of GHS and pattern of healthcare utilization across socioeconomic groups are two key measures. It is expected that every Chinese people not only gets financial protection but also has access to needed health care [28].

Healthcare services are prone to benefit inequity. Therefore, this study took healthcare services as research subject and examined the healthcare benefit incidence in different healthcare services across socioeconomic groups, such as primary health care, outpatient and inpatient services. Different from previous studies [21, 23, 29], primary care services were analyzed separately in our study, and fitted the equity target of GHS.

The analytic results found that the subsidies were pro-poor for primary care with negative CI values both in urban and rural populations. It indicated that poorer groups received a greater share of benefits from the use of primary services than richer groups [30], especially in rural population. Conversely, almost all CI values in both urban and rural areas were positive in outpatient and inpatient services, and the subsidies were pro-rich. It suggested that richer groups received a greater share of benefits from the use of outpatient and inpatient services in high-level facilities than poorer groups. Although the subsidies in outpatient and inpatient services were pro-rich, it presented progressivity in all rural and urban populations (KI values were all negative). The results showed that subsidies for the three healthcare services contributed to the narrowing of economic gap between the poor and rich and to the achievement of equity.

The different allocations of subsidies between primary care and outpatient and inpatient services might be attributed to various factors. The substantially increased spending on primary care is a key factor for benefit equity. Primary care facilities have played important roles in the new medical reform. Chinese government took steps to support primary healthcare facilities by increasing the governmental budget, providing technical support and qualified medical staff and so on. The poor management of primary healthcare facilities was reversed, and the service quality got improved. In addition, the reimbursement rates of public health insurance schemes at primary care facilities were higher than those at other levels of facilities in China. This policy encouraged patients, especially poor ones, to seek primary health services. To a certain extent, the above-mentioned facts changed the care-seeking behaviors, especially in rural populations.

As mentioned earlier, patients got benefits from GHS if they sought medical care at health care facilities. As study mentioned [23], primary healthcare facilities are most frequently visited by the poor. Government allocation of significant shares of their healthcare appropriations to primary health facilities widens access to medical care and improves healthcare utilization among rural patients. As shown in Table 4, the poor chose primary care more often than the rich did. Using urban area as an example, 27.41% of the poorest and 24.01% of the poorer sought primary health care, compared with 9.61% and 19.57 of the richest and richer, respectively.

**Table 4:** Healthcare utilization

	Income quintiles	Per capita household expenditure (%)	Primary health care (%)	Outpatient care (%)	Inpatient care (%)	Total (%)
Urban	Lowest quintile	5.65	27.41	14.64	14.59	20.45
	2	11.87	24.01	13.51	16.01	19.66
	3	17.37	19.4	19.59	15.59	17.34
	4	23.41	19.57	24.1	20.72	20.2
	Highest quintile	41.7	9.61	28.15	33.09	22.34
	Total	100	100	100	100	100
Rural	Lowest quintile	3.67	24.4	17.34	16.74	21.4
	2	10.97	23.26	10.89	14.13	19.69
	3	17.51	18.41	21.37	15.24	17.18
	4	23.88	15.96	19.35	15.66	15.85
	Highest quintile	43.97	17.97	31.05	38.23	25.88
	Total	100	100	100	100	100

Studies in low and middle income countries have shown that the poorest population tends to use primary care more than the rich [7, 15]. These results have led to calls for direct additional funds to primary care as a way of promoting equity. While these funds are expected to promote access to primary care for all, care should be taken to ensure that the poorest people, who bear the greatest burden of ill-health, continue to benefit from these services.

Different healthcare services has different unit subsidies, but the living standards are not considered. That is, rich and poor patients receive the same amount of subsidy for the same healthcare service. It is also a possible factor for inequity in GHS allocation. All along, there is a phenomenon in China that patients prefer to seek medical care at higher levels of hospitals, even for illnesses that could easily be treated at a primary care facility, despite of the high price and out-of-pocket costs. The wealthy are more able to afford high level healthcare services and therefore have more opportunities to obtain corresponding government health subsidies. As shown in Tables 2, 3, the wealthy shared more than half of the subsidies both in urban and rural populations.

There is widespread agreement that the benefits of health services should be allocated across a population according to individuals’ need for health care rather than on the basis of their ability to pay for care or place of residence [31]. The relationship between the allocations of healthcare need and benefits showed in Figs 4 and 5. In this study, we applied “self-reported illness and chronic disease” as the proxy of healthcare need. Previous studies have argued that self-reported illness can be a poor measure of health need [32–34]. Because the low-income groups tend to be lower ‘recognition’ of illness than higher income groups. This could be partially explained by the fact that the poor cannot ‘afford’ to be ill (either in terms of the opportunity cost of lost work time or due to poor health service access), while high-income groups are likely to have relatively good access to health services as well as sick leave benefits in their formal sector jobs [31, 33]. We included questions on self-assessed health status and on chronic illness. In view of the limitations of “self-reported illness”, our study may underreport the healthcare need, the gap between subsidy benefits and healthcare need is still large.

Currently, Urban Employee Basic Medical Insurance (UEI) and Urban & Rural Resident Basic Medical Insurance (URRI) have progressed toward universal insurance coverage in policy level. However, universal insurance coverage is not synonymous with universal health coverage [35], and equitable GHS distribution still faced some challenges. Firstly, high OOP payments may be a major challenge to the equity in GHS benefit distribution. High user fees decrease healthcare utilization, and the poor who need medical services but cannot afford them will be excluded from the benefits. What’s more, inequitable distribution of social determinants of health, disparities in income and wealth, differences in geography and natural environment between the urban and rural areas, between the eastern and western regions, and between households have widened substantially [36]. The growth in social determinants inequality could have damaged health effects. In addition, inefficient uses of medical resources lead to the situation that resources are not well distributed to where they would have the greatest health benefit. High-quality medical resources and government subsidies often skew toward high-level facilities, while low level provision of care get very little. With the deepening of the health system reform and the transformation of government functions in order to tackle the challenges described above, actions should be taken to: first, clear the goal of GHS, increase medical expenditures, and optimize GHS structure; second, skew GHS toward primary healthcare services and promote the sinking of high quality medical resources; third, focus on low-income people and take the way of “boosting the demand-side”; finally, improve the Hierarchical Medical System, regulate health seeking behavior and guide the reasonable behavior for medical treatment of residents.

There are some limitations of this study that should be mentioned. The classification of living standards was based on the household expenditures. Despite that expenditures have been recognized as a preferred measure of living standard [14], self-reported household expenditures might be inaccurate due to recall bias or deliberate underreporting. Similarly, this bias also exists in the information of healthcare utilization. In addition, our study assumed that the quality of the same type of service used in different regions or groups was the same. Ignoring the quality of healthcare services may also affect the results, but it is difficult to measure. What’s more, government allocated subsidies only into public medical facilities at all levels, therefore, residents cannot obtain GHS when they use healthcare services at private medical facilities. Moreover, like most BIA studies, we conducted what is called a “standard” BIA rather than a “marginal” BIA, some argued the latter is the more policy-relevant of the two exercises [37]. In addition, we did not have data from other China’s National Health Services Surveys outside of 2013, the impact of the GHS cannot be supported further with more robust outcome measures (eg. Changes in health outcomes since 2009).

## Conclusions

The inequitable distribution of GHS in China exited in different healthcare services; however, the GHS benefit was generally progressive. This inequity in healthcare benefit allocation is a marker of overall health system performance and progress towards achieving UHC. Future healthcare reforms in China should not only focus on expanding the coverage but also on improving the equity of distribution of healthcare benefits.

## Abbreviations

AE: Adult equivalents
BIA: Benefit incidence analysis
CH: Country hospital
CHS: Community health center
CI: Concentration index
DH: District hospital
GHS: Government Health Subsidy
KI: Kakwani index
MH: Municipal hospital
NHS: National health service
NHSS: National Health Services Survey
OOP: Out-of-pocket payment
PSU: Primary sampling unit
SHI: Social health insurance
SSU: Secondary sampling unit
THC: Township health center
UHC: Universal Health Coverage
VC: Village clinic
